# Long-term HLA-incompatible kidney transplant outcomes

**DOI:** 10.3389/ti.2026.16478

**Published:** 2026-07-16

**Authors:** Maria Kahanpää, Jouni Lauronen, Marko Lempinen, Kaisa Ahopelto, Ville Sallinen, Ilkka Helanterä

**Affiliations:** 1 Helsinki University Central Hospital, Helsinki, Finland; 2 Histocompatibility laboratory, Finnish Red Cross Blood Service, Helsinki, Finland

**Keywords:** acute rejection, antibody-mediated rejection, donor-specific antibodies, HLA incompatible, kidney transplantation

## Abstract

Many centers avoid human leukocyte antigen (HLA) -incompatible kidney transplantations due to increased risk of graft loss, although it may be the only option for highly sensitized patients. We assessed the association of pre-transplant donor-specific HLA-antibodies (DSAs) with biopsy-proven acute rejection (BPAR), antibody mediated rejection (AMR), and death-censored graft survival (DCGS). All deceased donor kidney transplantations in Finland between 2006 and 2021 were included (n = 3209), with 226 DSA-positive recipients. DSA characteristics, including specificity and mean fluorescence intensity (MFI), were collected. DSAs were associated with worse DCGS, higher risk of BPAR (HR 3.22, 95% CI 2.57–4.04), and especially AMR (HR 35.1, CI 22.4–55.1). Higher cumulative MFI was associated with lower 10-year DCGS (no-DSA 83%, ≥10,000 66%). A similar trend was seen in multivariable models (HR 1.81, CI 0.995–3.27 for MFI ≥10,000). Class II DSAs indicated higher risk for BPAR (HR 4.2 vs. HR 3.1), and AMR (HR 32.3 vs. HR 21.9), than class I DSAs. Collectively, pretransplant DSAs and higher cumulative MFI were associated with poorer graft survival, and especially class II DSAs were associated with higher risk of AMR. However, DCGS with even very high-level pretransplant DSA could be considered acceptable, supporting consideration of HLA-incompatible transplantations for highly sensitized patients.

## Graphical Abstract

**Figure:**
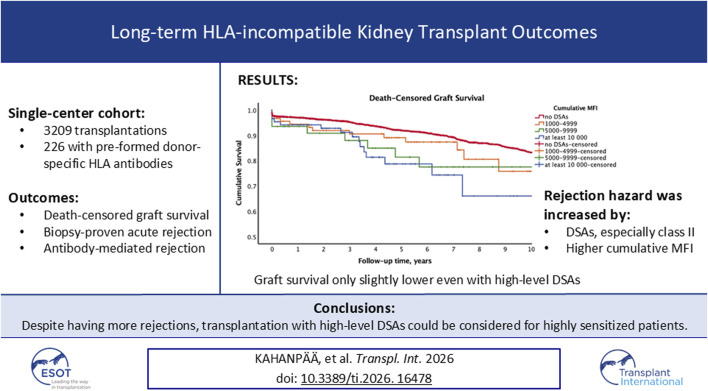

## Introduction

Although access to HLA-compatible transplantation has been increased in the recent years due to increased utilization of acceptable mismatch programs and living donor kidney paired exchange programs, [1, 2], HLA- incompatible (HLAi) transplantation remains the best alternative to get timely access to kidney transplantation for many very highly sensitized patients.

HLA- incompatible kidney transplantation can refer to the presence of pre-existing DSAs or to a positive flow cytometry or cytotoxic crossmatch, with an incremental increase in the risk of graft loss or early AMR [3]. In optimal circumstances with a living kidney donor, desensitization can be planned and performed pretransplantation. However, with deceased donor transplantation the options are scarce for rapid antibody removal. Recently, imlifidase has become available in some parts of the world (European Union, Australia), but only limited real-world experience exists until recently and high costs of the treatment limit the use of imlifidase to special circumstances [4].

The outcome of HLAi kidney transplantation is inferior compared to HLA compatible transplantation, and pre-existing DSAs (positive virtual crossmatch) are often avoided in organ allocation. On the other hand, the survival benefit of HLAi transplantation still exceeds survival while staying in dialysis [5]. Although many studies have shown the inferior graft survival and high risk of AMR associated with pre-existing donor-specific antibodies [6–8], many details of this association, e.g., with regard to what is the maximum level of DSAs still acceptable and what is the association for long-term survival beyond the first 10 years, have not been characterized adequately.

The aim of this nationwide cohort study is to characterize the association of pre-existing DSAs with long-term graft survival and the risk of AMR in detail, with special focus on those patients who have very high levels of DSAs pretransplantation, to be able to define a maximum threshold for pre-existing antibodies with still acceptable graft survival.

## Materials and methods

### Data

This study was a retrospective analysis of all adult kidney-only transplantations in Finland between January 2006 and December 2021. The data were obtained from the national Finnish Transplant Registry, and/or electronic patient records from Helsinki University Hospital (HUS), which is the only transplant center in Finland. HLA antibody analyses were made at the Finnish Red Cross Blood Service, which process this data on behalf of HUS. This study was approved by the Institutional Review Board of HUS, Abdominal Center (HUS/136/2024)

**FIGURE 1:**
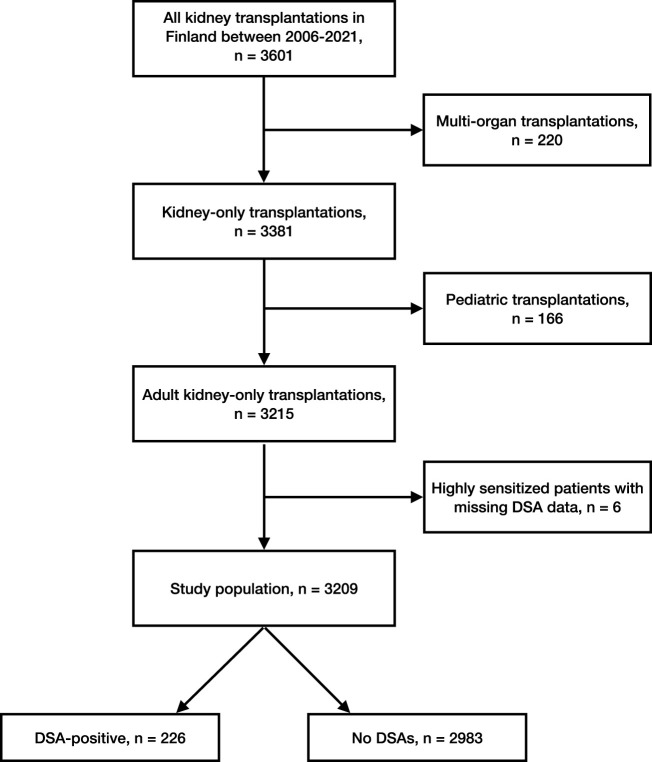
Flow chart presenting the inclusion and exclusion criteria of the patient material.

The inclusion and exclusion criteria of the patient material are presented in Figure 1. Living donor transplants and multi-organ transplantations were excluded. All transplantations were from donors after brain death, as donors after circulatory death were not used in our center during the study period. All transplantations were complement dependent cytotoxicity crossmatch negative, examined with donor splenocytes between 2006 and 2015 and peripheral T- and B-cells thereafter. No desensitization was used. According to the local immunosuppression protocol, the baseline immunosuppression was a combination of a calcineurin inhibitor (cyclosporine or tacrolimus, of which cyclosporine was used in earlier years, and tacrolimus increasingly in the later years), mycophenolate (1,000 mg twice daily with cyclosporine or 500 mg twice daily with tacrolimus) and steroids. Induction was with basiliximab for recipients with retransplantations or HLA mismatch grade over 3 in the A, B, and 1 DR loci. Anti-T-lymphocyte globulin was used for induction after 2015 in patients with known or presumed (panel reactive antibody (PRA) percentage over 80%) DSAs at the time of transplantation. Target trough levels for tacrolimus are 7–10 μg/L for the first 3 months and later 4–6 μg/L. As for cyclosporine, target levels are 160–200 μg/L for the first 3 months and 80–110 μg/L until 1 year after transplantation. Steroids were withdrawn after the first posttransplant year for most stable patients with no history rejections or immunological baseline kidney disease.

Data of the recipients included number of previous kidney transplants, time on the waitlist, age at transplantation, sex, height, weight, body-mass index, ABO blood type, smoking status, ICD-10 code of kidney disease, cytomegalovirus (CMV) status, highest and last calculated PRA percentage for class I and II HLAs pre-transplantation, dialysis time and last dialysis type (hemodialysis or peritoneal dialysis). Donor data included gender, age, height, weight and CMV status. Transplantation data included HLA AB and DR mismatch grades, cold ischemia time, follow-up time and status of graft survival, biopsy-proven acute rejection (yes or no), and rejection time and type (T-cell or antibody mediated), as categorized by the respective Banff classification at the time of biopsy [9]. Borderline findings were registered as rejections if they were treated accordingly. Mixed phenotypes were classified as AMR.

The DSA status of sensitized patients, including specificity and MFI, were collected from posttransplant tissue compatibility reports. One Lambda Labscreen® mixed and single antigen beads with Luminex® were used for HLA antibody screening and identification with the use of HLA Fusion software (One Lambda Inc., Canoga Park, CA). MFI level >1,000 was considered positive. Highly sensitized recipients with no available DSA data (n = 7) were excluded from the study. Donor HLA typing was performed at intermediate resolution, complemented with haplotype analyses.

### Analyses

Recipients were compared in different groups based on their DSA status (DSAs vs. no DSAs). Recipients with DSAs were also divided into different subgroups based on their cumulative overall DSA MFI: 1,000–4,999, 5,000–9,999, or at least 10,000. To find out the significance of class I vs. class II DSAs, the DSA group was also categorized based on the separate class I and class II cumulative MFIs, using the same above-mentioned limits. DSA MFI was also tested for the models as a continuous variable, but as the linearity assumption was not met, categorized MFI was used instead.

**FIGURE 2:**
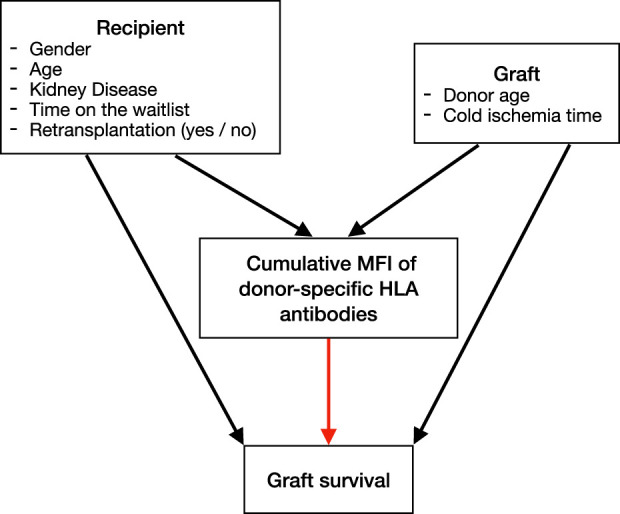
Factors possibly affecting the presence of DSAs at the time of transplantation and associating with graft survival.

The outcomes examined included DCGS, defined as return to dialysis or retransplantation, and censoring for deaths; biopsy-proven acute rejections (BPAR) and AMR. For multivariable Cox regression analyzing factors associated with DCGS, confounder analysis for the model was constructed as a directed acyclic graph (DAG) [10]. The DAG (Figure 2) presents factors possibly affecting the presence of DSAs at the time of transplantation and confounding the associations with graft survival. For Cox regression models, we included cumulative MFI (categorized into four groups (no DSAs, 1,000–4,999, 5,000–9,999, at least 10,000)), donor age, recipient sex, age and kidney disease (diabetic kidney disease, glomerulonephritis, polycystic kidney disease or other), retransplantation (yes or no), time on the waitlist, cold ischemia time, use of induction immunosuppression (other than iv steroids), and use of cyclosporine (vs. tacrolimus), as covariates. Relevant first-degree interactions between DSAs and other variables were tested, and the assumption for proportional hazard was supported for DSAs. In addition, interaction between retransplantation and cumulative MFI, was examined (and found nonsignificant). Graft survival was estimated with the Kaplan-Meier method where graft loss (return to dialysis or retransplantation) was used as the event while death and end of follow-up time were censored. Factor comparisons for DCGS were calculated with the Log Rank test.

For multivariable analysis of BPAR and AMR, a Cox regression analysis was run with the same covariates as the analysis regarding DCGS, presented in the DAG, taking rejection time into account. Interaction between retransplantation and cumulative MFI was examined and found insignificant for BPARs and significant for AMRs. To compare the risk of rejections within different groups, univariable analyses were calculated using Cox regression, for DSA status (any DSAs, and class I and class II DSAs separately), and categorized cumulative MFI for total MFI, and class I and class II separately. The assumption for proportional hazard was again supported for DSAs.

As a sensitivity analysis, all statistical tests were repeated for immunodominant MFIs for class I, class II and all DSAs.

All statistical analyses were done using IBM SPSS Statistics, version 28.0. P-value of under 0.05 was considered statistically significant. Missing data were assumed to be missing at random, and as the number of missing data were low for the variables of interest for the multivariable analyses, cases with missing values were excluded from the respective analyses.

## Results

### Study population

A total of 3209 transplantations were included in this study, of which 226 (7%) recipients were DSA positive at the time of transplantation. The patient characteristics are described in Table 1. The only missing data were for recipient BMI (n = 361), time on the waitlist (n = 4), recipient CMV status (n = 29), donor CMV status (n = 53), donor age (n = 1), cold ischemia time (n = 2), last higher and total PRA% before transplantation (n = 294), average maximum PRA% before transplantation (n = 238), HLA AB mismatch (n = 1) and DR mismatch (n = 1). Statistically significant differences were found for recipient sex, retransplantation, baseline kidney disease, recipient time on waitlist, recipient CMV positivity, donor age, being on tacrolimus (vs. cyclosporine), induction immunosuppression, delayed graft function and HLA AB mismatch. The maximum follow-up time was 16.0 years, with a median of 5.4 years.

**TABLE 1:** Patient characteristics in recipients with and without DSAs.

Patient characteristic	DSA (n = 226)	No DSA (n = 2,983)
Recipient age, median (IQR), years	53 (21)	54 (19)
Recipient sex, male	100 (44%)	1968 (66%)
Recipient BMI, median (IQR), kg/m2	24.48 (5.8)	25.31 (6.2)
Retransplantation	120 (53%)	265 (8.9%)
Baseline kidney disease Diabetic kidney disease Glomerulonephritis Polycystic kidney disease Other	28 (12%) 75 (33%) 36 (16%) 87 (39%)	838 (28%) 746 (25%) 530 (18%) 869 (29%)
Recipient time on waitlist, median, years	2.2 (2.5)	0.38 (0.70)
Recipient CMV positive	181 (80%)	2,176 (74%)
Donor CMV positive	175 (79%)	2,304 (79%)
Donor age, median (IQR), years	59 (18)	56 (19)
Cold ischemia time, median (IQR), hours	19 (8.4)	19 (8.1)
On tacrolimus (vs. cyclosporine)	162 (72%)	1,254 (42%)
Induction immunosuppression (other than iv steroids) Basiliximab Anti-thymocyte globulin	67 (61%) 77 (34%) 60 (27%)	633 (21%) 397 (13%) 236 (8%)
Delayed graft function	83 (37%)	831 (28%)
Last higher PRA% (class I or II) before transplantation, median (IQR)	91 (25)	0 (8)
Last PRA% (class I and II) in total before transplantation, median (iQR)	116 (95)	0 (8)
Average maximum PRA% before transplantation, median (IQR)	71 (47)	0 (11)
HLA AB mismatch, median (IQR)	2 (1)	2 (2)
HLA DR mismatch, median (IQR)	1 (0)	1 (1)

Among recipients with DSAs, the mean cumulative MFI was 11,563 (SD 13 886). Altogether 90 recipients had a cumulative MFI of 1,000–4,999, 46 had 5,000–9,999, and 86 had at least 10,000. Four DSA positive recipients did not have the complete MFI data available, of which three recipients had only class I DSAs with missing MFI, and one recipient had both class I and II DSAs with MFI available for class I but unavailable for class II, and these recipients were excluded from the analyses concerning missing data.

Altogether 162 recipients had class I DSAs and 119 had class II DSAs, while 55 recipients had both. Categorized into groups based on class I DSAs, 77 recipients had a cumulative MFI of 1,000–4,999, 43 had 5,000–9,999 and 40 had at least 10,000. As for class II DSAs, 48 patients had a cumulative MFI of 1,000–4,999, 25 had 5,000–9,999, and 45 had at least 10,000.

### Graft survival

**FIGURE 3:**
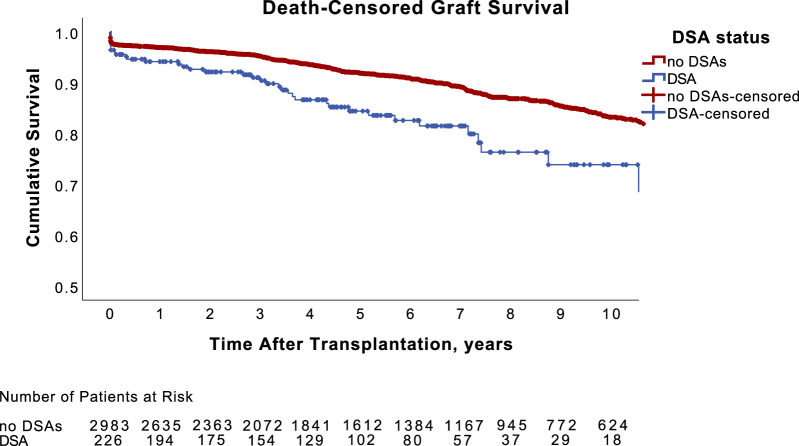
Death-censored graft survival in recipients with and without DSAs.

DSAs were associated with worse death-censored graft survival (p < 0.001) in comparison to no DSAs (Figure 3). Recipients with DSAs had a 5-year DCGS of 84% and 10-year survival of 74% while recipients without DSAs had a 92% 5-year DCGS and an 83% 10-year DCGS.

**FIGURE 4:**
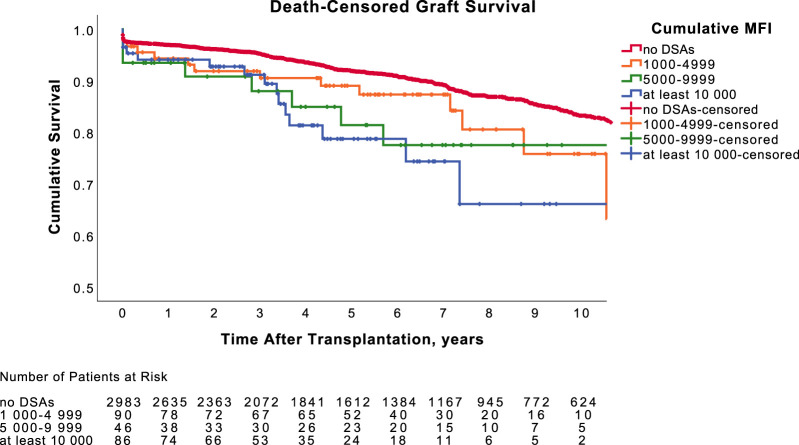
Death-censored graft survival in recipients without DSAs and with different cumulative MFI levels of DSAs.

Higher cumulative MFI indicated worse 5-year DCGS (no-DSA: 92%, MFI 1,000–4,999: 89%, 5,000–9,999: 81%, at least 10,000: 79%, p < 0.001) and 10-year graft survival (no-DSA 83%, 1,000–4,999: 76%, 5,000–9,999: 78%, at least 10,000: 66%) (Figure 4). However, there were no statistically significant differences between the groups with different cumulative MFI levels (p = 0.441).

The results of multivariable Cox regression analysis regarding DCGS are presented in Table 2. Recipient age, baseline kidney disease, cold ischemia time, donor age and retransplantation were independently associated with DCGS. A trend was seen in the association of categorized cumulative MFI with worse DCGS, but this association did not reach statistical significance.

**TABLE 2:** Multivariable Cox regression analysis for DCGS.

Variable	Hazard ratio (95% CI)	p-value
Baseline kidney disease (vs. diabetic kidney disease) Glomerulonephritis Polycystic kidney disease Other	1.050 (0.796–1.384) 0.613 (0.429–0.876) 0.917 (0.693–1.213)	0.019 0.732 0.007 0.543
Categorized cumulative MFI (vs. no DSAs) 1,000–4,999 5,000–9,999 At least 10,000	1.441 (0.820–2.530) 1.722 (0.839–3.535) 2.024 (1.096–3.737)	0.087 0.204 0.138 0.024
Cold ischemia time (for each 1 min increased)	1.001 (1.000–1.001)	<0.001
Donor age (for each 1 year increased)	1.025 (1.016–1.034)	<0.001
Induction immunosuppression, other than iv steroids (vs. no)	0.787 (0.569–1.087)	0.146
On cyclosporine (vs. tacrolimus)	1.235 (0.952–1.601)	0.112
Recipient age (for each 1 year increased)	0.985 (0.977–0.993)	<0.001
Recipient sex (vs. female)	0.952 (0.767–1.181)	0.655
Retransplantation (vs. no)	1.418 (1.002–2.008)	0.049
Time on the waitlist (for each 1 day increase)	1.000 (1.000–1.000)	0.334

**FIGURE 5:**
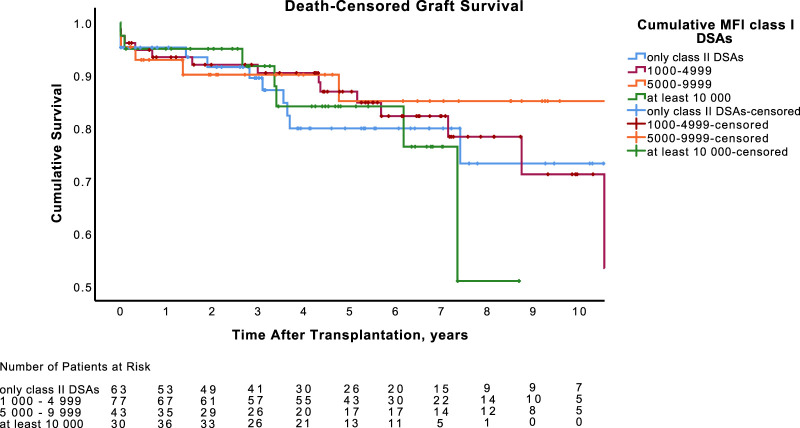
Death-censored graft survival with different cumulative MFI levels of class I DSAs.

**FIGURE 6:**
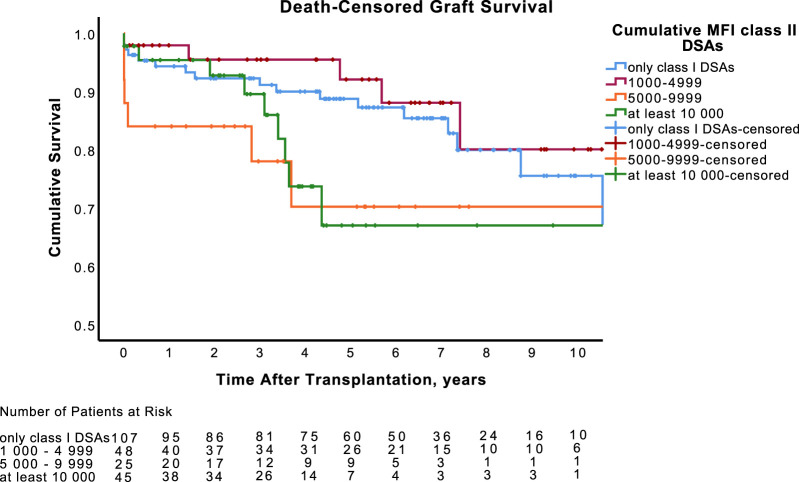
Death-censored graft survival with different cumulative MFI levels of class II DSAs.

Regarding class I DSAs, there was no statistically significant (p = 0.841) association of higher cumulative MFI indicating worse 5-year DCGS or 10-year DCGS (Figure 5). Similarly, regarding class II DSAs, there was no statistically significant (p = 0.069) association of higher cumulative MFI indicating worse 5-year DCGS or 10-year DCGS (Figure 6).

### Acute rejections

Rejection frequencies for BPAR, AMR and TCMR are described in Table 3. Hazard ratios for BPAR and AMR are presented in supplementary material, Supplementary Table S1.

**TABLE 3:** Biopsy-proven acute rejection, antibody-mediated rejection and T-cell mediated rejection frequencies within different comparison groups.

Group (n)	BPARs (%)	AMRs (%)	TCMRs (%)
No DSAs vs. DSAs No DSAs (2,983) DSAs (226)	474 (16) 90 (40) p = 0.000	28 (0.94) 59 (26) p = 0.000	447 (15) 32 (14) p = 0.737
Cumulative MFI 1,000–4,999 (90) 5,000–9,999 (46) At least 10,000 (86)	27 (30) 16 (35) 45 (52) p = 0.008	11 (12) 11 (24) 35 (41) p < 0.001	16 (18) 6 (13) 10 (12) p = 0.488
Class I DSAs: Cumulative MFI no class I DSAs (63) 1,000–4,999 (77) 5,000–9,999 (43) At least 10,000 (40)	26 (41) 23 (30) 18 (42) 22 (55) p = 0.067	17 (27) 12 (16) 15 (35) 14 (35) p = 0.0499	10 (16) 11 (14) 3 (7.0) 8 (20) p = 0.382
Class II DSAs: Cumulative MFI no class II DSAs (107) 1,000–4,999 (48) 5,000–9,999 (25) At least 10,000 (45)	33 (31) 21 (44) 14 (56) 21 (47) p = 0.057	18 (17) 10 (21) 12 (48) 18 (40) p < 0.001	15 (14) 11 (23) 3 (12) 3 (6.7) p = 0.159

DSAs were associated with more BPAR (HR 2.0, 95% CI 1.6–2.5, p < 0.001), with even higher HR for acute AMR (HR 22.7, 95% CI 14.3–36.2, p < 0.001), as 40% of DSA positive recipients had BPARs and 26% had AMR. Comparing only the class I or class II DSA status to those without the above-mentioned DSAs, both acute overall rejections (class I 39.5% vs. class II 47.9%) and acute AMR (class I 25.9% vs. class II 34.5%) were more prevalent in recipients with class II DSAs, but the HRs were higher for class I. Comparing recipients with class I DSAs to those without them, HR for BPAR was 2.2 (95% CI 1.7–2.9, p < 0.001) and HR for acute AMR was 16.4 (95% CI 10.5–25.6, p < 0.001), while comparing recipients with class II DSAs to those without them, the HR for BPAR was 1.8 (95% CI 1.4–2.4, p < 0.001) and the HR for acute AMR was 13.9 (95% CI 9.0–21.4, p < 0.001).

Examining the cumulative effect of DSAs, both the hazard ratio of BPAR (p < 0.001) and acute AMR (p < 0.001) was increased with the increasing cumulative MFI, categorized into 1,000–4,999, 5,000–9,999 and at least 10,000. Regarding cumulative class I MFIs, the frequency of acute AMR (p = 0.0499) was increased with the increasing categorized cumulative MFI. There was no statistically significant difference for the frequency of BPAR, but the HR increased for every MFI limit for both BPAR and AMR. As for different categories of cumulative class II MFIs, the frequency of BPAR (p = 0.057) and AMR (p < 0.001) was decreased at the limit of 10,000, which was also seen in hazard ratios at the same limit.

The multivariable Cox regression analyses for the risk of acute rejections using the categorized cumulative total MFI are presented in Table 4. For acute rejection, statistically significant risk factors were categorized cumulative MFI, donor age, induction immunosuppression, and being on cyclosporine vs. tacrolimus recipient age, while categorized kidney disease, cold ischemia time, recipient sex, retransplantation and time on the waitlist were statistically unsignificant. Regarding acute AMR, the only statistically significant risk factors were categorized cumulative MFI and recipient age.

**TABLE 4:** Results of multivariable Cox analysis regarding BPARs and AMRs.

Variable	BPARs, hazard ratio (95% CI)	BPARs, p-value	AMRs, hazard ratio (95% CI)	AMRs, p-value
Categorized cumulative MFI (vs. no DSAs) 1,000–4,999 5,000–9,999 At least 10,000	1.943 (1.288–2.929) 2.311 (1.352–3.951) 4.505 (3.041–6.674)	<0.001 0.002 0.002 <0.001	9.342 (4.325–20.178) 15.769 (6.865–36.218) 35.884 (16.723–77.001)	<0.001 <0.001 <0.001 <0.001
Categorized kidney disease (vs. diabetic kidney disease) Glomerulonephritis Polycystic kidney disease Other	0.892 (0.704–1.129) 0.748 (0.565–0.989) 0.944 (0.756–1.177)	0.217 0.341 0.042 0.607	1.512 (0.693–3.299) 1.422 (0.574–3.521) 1.848 (0.869–3.932)	0.430 0.299 0.447 0.111
Cold ischemia time (for each 1 min increased)	1.000 (1.000–1.000)	0.953	1.000 (1.000–1.001)	0.349
Donor age (for each 1 year increased)	1.022 (1.014–1.029)	<0.001	1.017 (1.000–1.035)	0.056
Induction immunosuppression, other than iv steroids (vs. no)	0.771 (0.618–0.962)	0.021	1.443 (0.852–2.441)	0.172
On cyclosporine (vs. tacrolimus)	0.603 (0.499–0.729)	<0.001	0.618 (0.360–1.061)	0.081
Recipient age (for each 1 year increased)	0.981 (0.974–0.988)	<0.001	0.972 (0.956–0.989)	0.001
Recipient sex (v. female)	1.186 (0.991–1.419)	0.063	0.994 (0.633–1.562)	0.980
Retransplantation (v. no)	0.953 (0.723–1.255)	0.730	0.922 (0.501–1.695)	0.793
Time on the waitlist (for each 1 day increased)	1.000 (1.000–1.000)	0.168	1.000 (1.000–1.001)	0.065

### Sensitivity analyses

As a sensitivity analysis, all analyses were repeated for immunodominant MFIs for class I, class II and all DSAs. The results were considered similar to the primary analyses, as higher MFI for the immunodominant DSA was similarly associated with increased risk of rejection and inferior graft survival in all analyses. The results of these sensitivity analyses are presented in supplementary material, Supplementary Tables S2, S3 and Supplementary Figures S1–S3.

## Discussion

Our aim was to evaluate our national transplant center’s long-term experience with HLA incompatible deceased donor transplantations. As has been described previously, DSAs were associated with worse graft survival, and especially cumulative MFI of DSAs was associated with worse prognosis. However, we were able to show that this association did not reach statistical significance in multivariable models in long-term follow-up of up to 10 years. Although our findings may be confounded by multiple known or unknown factors, this finding suggests that the independent association of donor-specific antibodies with long-term graft survival may be less prominent than has been previously described. Importantly, we were able to show that long-term graft survival was acceptable even with the highest DSAs. For many of these highly sensitized patients, HLA compatible transplantation is not a realistic option without extensive waiting time, and the outcome of these HLAi transplantation should be compared with patients on dialysis. Furthermore, longer time on dialysis before transplantation is associated with worse survival [11], suggesting that some patients may benefit from HLA incompatible transplantation instead of waiting long periods on dialysis for an HLA compatible transplant.

Although graft survival was comparable after HLAi transplantation, the risk of acute rejection, especially antibody-mediated rejection was high, as expected. However, most of the rejections were reversible with standard treatment (iv steroids for TCMR and plasma exchange and IvIG for AMR), and only 4 grafts were lost due to irreversible antibody-mediated rejection.

There are several previous studies, which show that pre-existing DSAs are associated with worse graft survival and higher risk of AMR [12–14]. Especially higher MFI antibodies and class II antibodies have been associated with the risk of AMR [15]. In addition, persistent presence of DSA has been associated with worse outcomes [16], and especially class II DSAs seem to persist more frequently compared to class I DSAs [17]. Our findings are in line with previous studies, as also in our cohort class II DSAs seemed to be associated with even higher risk of AMR after transplantation. Regarding the intensity of DSAs, there is little previous data on recipients with as high DSA MFI levels as in our study, and the threshold limits for the analyses have been set significantly lower [13, 18]. Some studies have reported on more rejections and worse graft survival at the MFI level of 10,000 [19] or 11,000 [15], but the study samples have remained very small for crossmatch negative transplantations. In addition, in the current study follow-up times are long, even up to >10 years. Data regarding persistence of DSAs were, unfortunately, not available in our cohort.

Our novel findings of the relatively good long-term results in HLA-incompatible transplantation have several clinical implications. First, although acceptable mismatch programs (such as Scandiatransplant Acceptable Mismatch Program [1]) or paired exchange programs [2] provide the best alternative to find HLA compatible transplantations for many patients, some very highly sensitized patients have extremely low likelihood of finding an HLA compatible transplantation. Our findings suggest that for patients unable to get timely access to HLA compatible transplantation, HLA incompatible transplantation may be an acceptable alternative compared to dialysis, depending on the individual patient status. This may be true even in patients with high-level DSAs with negative cytotoxic crossmatch. One purpose of the current study was to identify thresholds or characteristics for unacceptable DSAs, e.g., to identify the most suitable patients for desensitization treatments (such as imlifidase). However, no MFI cut-offs or specific characteristics (such as class I or class II) could be identified, as even patients with the highest DSA MFI levels had acceptable outcomes.

Our current study has some limitations. The findings are from a single center, and they cannot be directly generalized to other cohorts, as the patient characteristics, and allocation, immunosuppression and rejection treatment protocols may vary from other regions. In addition, even though the long study period of 16 years could be considered a strength of this study, it includes some changes in our own immunosuppression protocols, most importantly the increasing use of tacrolimus in comparison to cyclosporine. However, no other major changes have occurred during this time span. The graft biopsy scoring and rejection criteria have also changed in accordance with the evolution of the Banff Classification [9], which could affect the generalizability of the results.

Another limitation is that DSAs were not always known at the time of transplantation, as for some patients the pretransplant DSA status was determined only after transplantation, not prospectively. Therefore, immunosuppression has not always been modified accordingly, and as a result only 61% of the patients with DSA received induction, and only 27% received lymphocyte-depleting induction. However, our findings suggest that graft survival with DSAs is acceptable even without heavy lymphocyte-depleting induction therapy, which increases the risk for leukopenia, infections, and malignancies after transplantation. Flow cytometry crossmatch is not in routine use in our country, so no data regarding the flow cytometry crossmatch results for the study cohort were available. Another limitation is the retrospective nature of this study, and causality cannot be concluded. On the other hand, a strength of our study is the large uniform cohort with long-term follow-up, including patients with very high levels of DSA at the time of transplantation, showing the natural course and good outcomes up to 10 years after transplantation.

In multivariable models, statistical significance was not always achieved, which could be related to limited statistical power or residual confounding. It is also debatable how strongly pre-transplant DSAs and acute rejections relate to long-term graft survival, although our results illustrate a consistent trend of the association. The ultimate decision-making of HLA incompatible transplantations with high-level DSAs should, however, be based on individual risk and benefit assessment.

## Conclusion

In conclusion, although pretransplant DSA and higher cumulative MFI of DSA were associated with worse kidney graft survival, the association did not remain significant in all models after adjustment with confounding factors. Long-term graft survival among patients even with high-level pretransplant DSAs could be considered acceptable in comparison to prolonged dialysis treatment, and these transplantations could be considered for highly sensitized patients as the last option, especially when desensitization is unavailable or contraindicated. DSAs, however, especially class II DSAs are associated with a high risk of antibody-mediated rejection, which should be taken into consideration when balancing risks and benefits of HLA incompatible transplantation.

## Data Availability

The datasets presented in this article are not readily available because According to Finnish law, sharing individual-level data outside the research personnel is prohibited (Act on Secondary Use of Health and Social Data, 2019). Requests to access the datasets should be directed to ilkka.helantera@helsinki.fi.

## Supplementary material

SUPPLEMENTARY FIGURE S1 Death-censored graft survival in recipients without DSAs and with different immunodominant MFI levels of DSAs, p < 0.001.

SUPPLEMENTARY FIGURE S2 Death-censored graft survival with different immunodominant MFI levels of class I DSAs.

SUPPLEMENTARY FIGURE S3 Death-censored graft survival with different immunodominant MFI levels of class II DSAs.

## References

[1] Weinreich I Bengtsson M Lauronen J Naper C Lokk K Helanterä I Scandiatransplant acceptable mismatch program – 10 years with an effective strategy for transplanting highly sensitized patients. Am J Transpl (2022) 22(12):2869–79. 10.1111/ajt.17182 36030513 PMC10087587

[2] Weinreich ID Andersson T Andrésdóttir MB Bengtsson M Biglarnia A Bistrup C Scandiatransplant exchange program (STEP): development and results from an international kidney exchange program. Transpl Direct (2023) 9:11. 10.1097/TXD.0000000000001549 PMC10581625 37854025

[3] Mamode N Bestard O Claas F Furian L Griffin S Legendre C European guideline for the management of kidney transplant patients with HLA antibodies: by the European Society for organ transplantation working group. Transpl Int (2022) 35:10511. 10.3389/ti.2022.10511 36033645 PMC9399356

[4] Furian L Heeman U Bengtsson M Bestard O Binet I Böhmig GA Desensitization with imlifidase for HLA-incompatible deceased donor kidney transplantation: a Delphi international expert consensus. Transpl Int (2025) 37:13886. 10.3389/ti.2024.13886 39867871 PMC11758882

[5] Orandi BJ Luo X Massie AB Garonzik-Wang JM Lonze BE Ahmed R Survival benefit with kidney transplants from HLA-incompatible live donors. N Engl J Med (2016) 374(10):940–50. 10.1056/NEJMoa1508380 26962729 PMC4841939

[6] Hariharan S Israni AK Danovitch G . Long-term survival after kidney transplantation. N Engl J Med (2021) 385:729–43. 10.1056/NEJMra2014530 34407344

[7] Caner S Döhler B Opelz G . Presensitized kidney graft recipients with HLA class I and II antibodies are at increased risk for graft failure: a collaborative transplant study report. Hum Immunol (2009) 70(8):569–73. 10.1016/j.humimm.2009.04.013 19375472

[8] Kardol-Hoefnagel T Senejohnny DM Kamburova EG Wisse BM Reteig L Gruijters ML Determination of the clinical relevance of donor epitope-spesific HLA-antibodies in kidney transplantation. HLA (2024) 103:1. 10.1111/tan.15346 38239046

[9] Roufosse C Simmonds N Clahsen-van Groningen M Haas M Henriksen KJ Horsfield C A 2018 reference guide to the banff classification of renal allograft pathology. Transplantation (2018) 102(11):1795–814. 10.1097/TP.0000000000002366 30028786 PMC7597974

[10] Suttorp MM Siegerink B Jager KJ Zoccali C Dekker FW . Graphical presentation of confoundingin directed acyclic graphs. Nephrol Dial Transpl (2015) 30(9):1418–23. 10.1093/ndt/gfu325 25324358

[11] Helanterä I Salmela K Kyllönen L Koskinen P Grönhagen-Riska C Finne P . Pretransplant dialysis duration and risk of death after kidney transplantation in the current era. Transplantation (2014) 98(4):458–64. 10.1097/TP.0000000000000085 24646770

[12] Mohan S Palanisamy A Tsapepas D Tanriover B John Crew R Dube G Donor-specific antibodies adversely affect kidney allograft outcomes. J Am Soc Nephrol (2012) 23(12):2061–71. 10.1681/ASN.2012070664 23160511 PMC3507372

[13] Ziemann M Altermann W Angert K Arns W Bachmann A Bakchoul T Preformed donor-specific HLA antibodies in living and deceased donor transplantation: a multicenter study. Clin J Am Soc Nephrol (2019) 14(7):1056–66. 10.2215/CJN.13401118 31213508 PMC6625630

[14] Michielsen LA Wisse BW Kamburova EG Verhaar MC Joosten I Allebes WA A paired kidney analysis on the impact of pre-transplant anti-HLA antibodies on graft survival. Nephrol Dial Transpl (2019) 34(6):1056–63. 10.1093/ndt/gfy316 30365008

[15] Malheiro J Tafulo S Dias L Martins LS Fonseca I Beirão I Analysis of preformed donor-specific anti-HLA antibodies characteristics for prediction of antibody-mediated rejection in kidney transplantation. Transpl Immunol (2015) 32(2):66–71. 10.1016/j.trim.2015.01.002 25661873

[16] Senev A Callemeyn J Lerut E Emonds MP Naesens M . Histological picture of ABMR without HLA-DSA: temporal dynamics of effector mechanisms are relevant in disease reclassification. Am J Transpl (2019) 19(3):954–5. 10.1111/ajt.15234 30582268

[17] Redondo-Pachón D Pérez-Sáez MJ Mir M Gimeno J Llinás L García C Impact of persistent and cleared preformed HLA DSA on kidney transplant outcomes. Hum Immunol (2018) 79(6):424–31. 10.1016/j.humimm.2018.02.014 29524568

[18] Debiais-Deschamps C Amrouche L Rabant M Duong Van Huyen J-P Burger C Roger C Long-term graft and patient survival in kidney transplant recipients with high levels of preformed DSAs (MFI > 3000): a propensity score-matched analysis. Transpl Direct (2025) 11:12. e1868. 10.1097/TXD.0000000000001868 41209480 PMC12594297

[19] Gloor JM Winters JL Cornell LD Fix LA DeGoey SR Knauer RM Baseline donor-specific antibody levels and outcomes in positive crossmatch kidney transplantation. Am J Transpl (2010) 10(3):582–9. 10.1111/j.1600-6143.2009.02985.x 20121740

