# Supplementary figures and images for: Long-term HLA-incompatible kidney transplant outcomes
Source: Transpl Int. 2026 Jul 16;39:16478. doi: 10.3389/ti.2026.16478 (PMC13422208; doi:10.3389/ti.2026.16478)

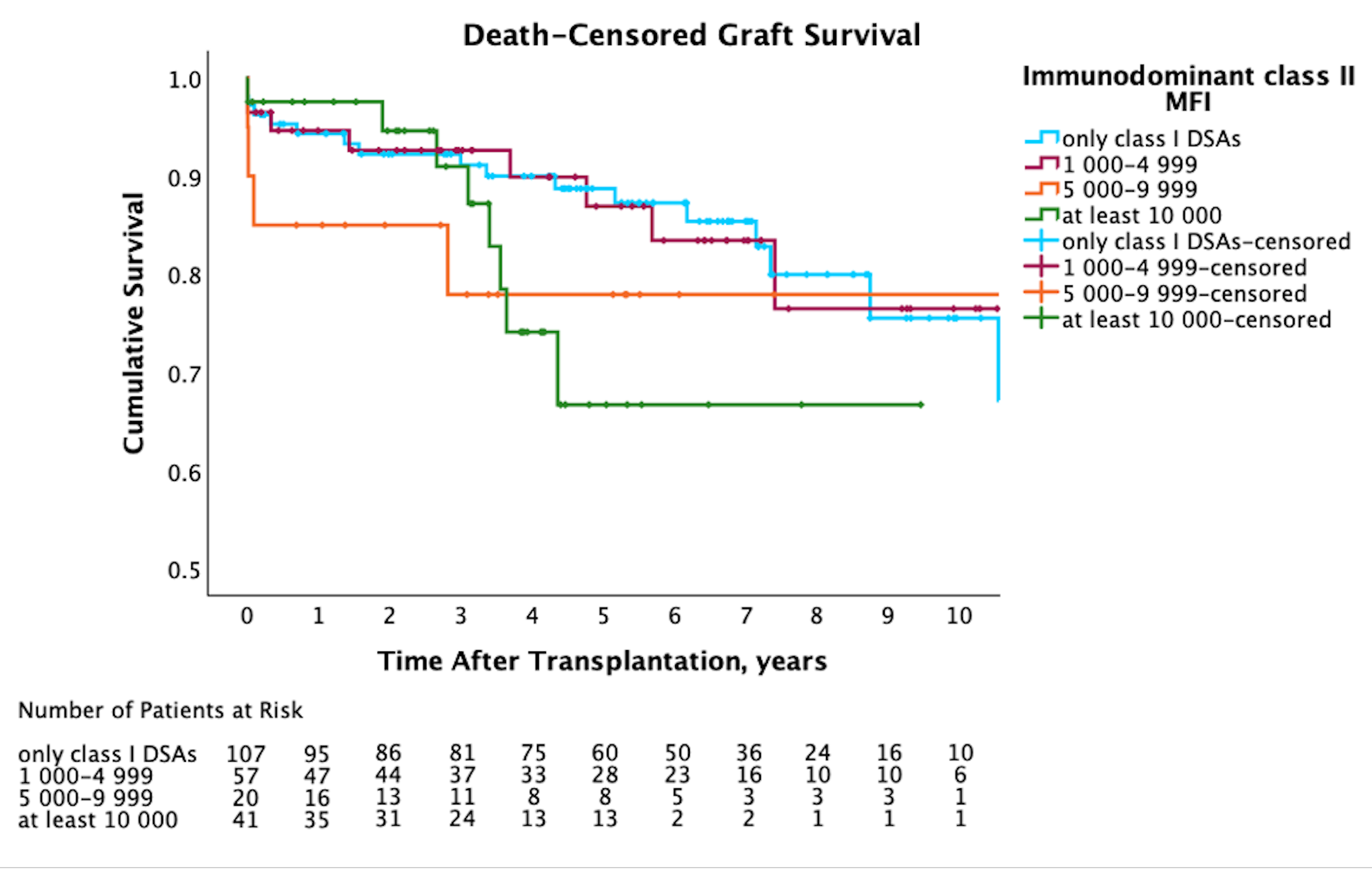

Supplement: Supplementary file 1 [file Image3.tiff]

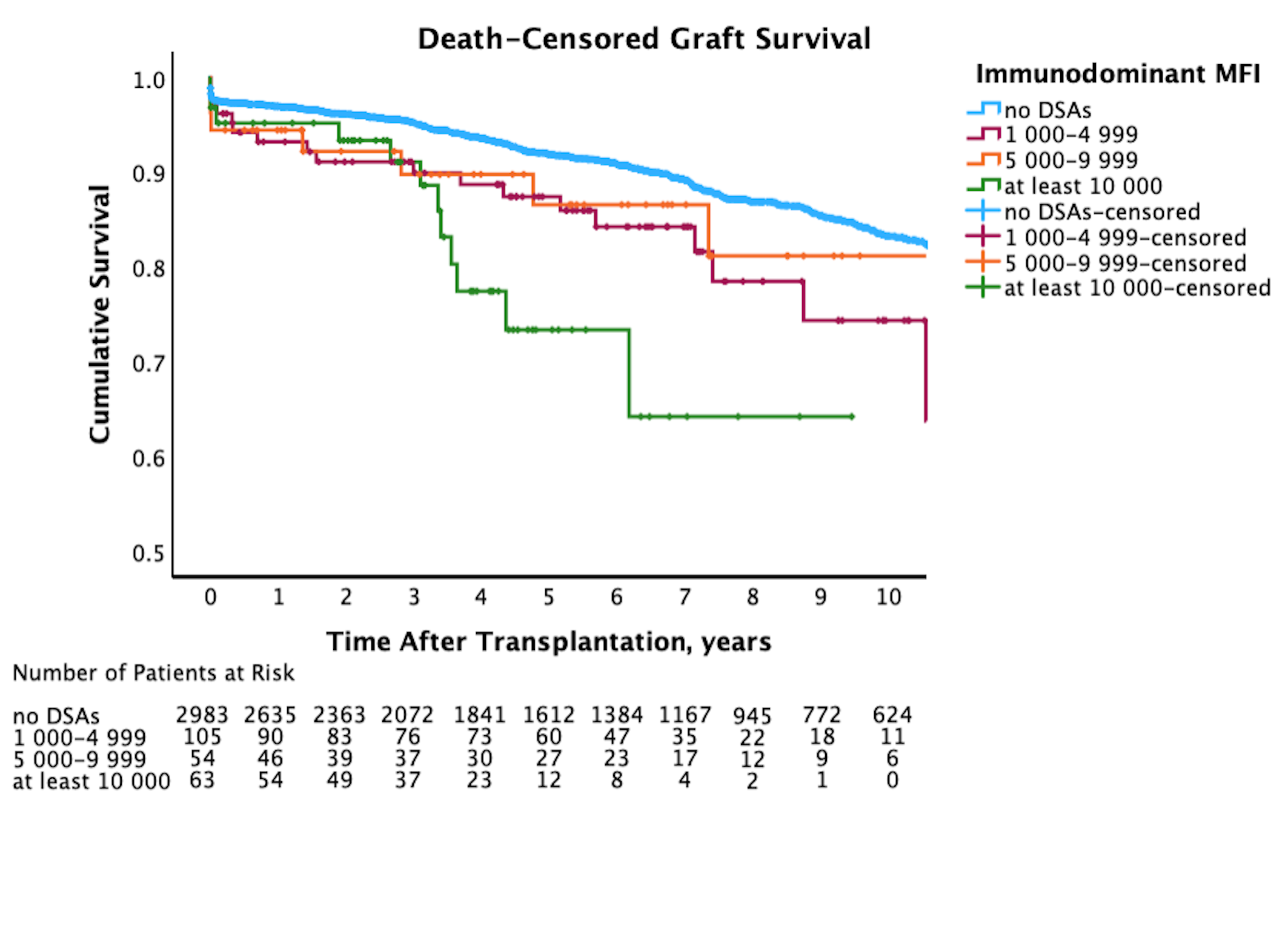

Supplement: Supplementary file 2 [file Image1.tiff]

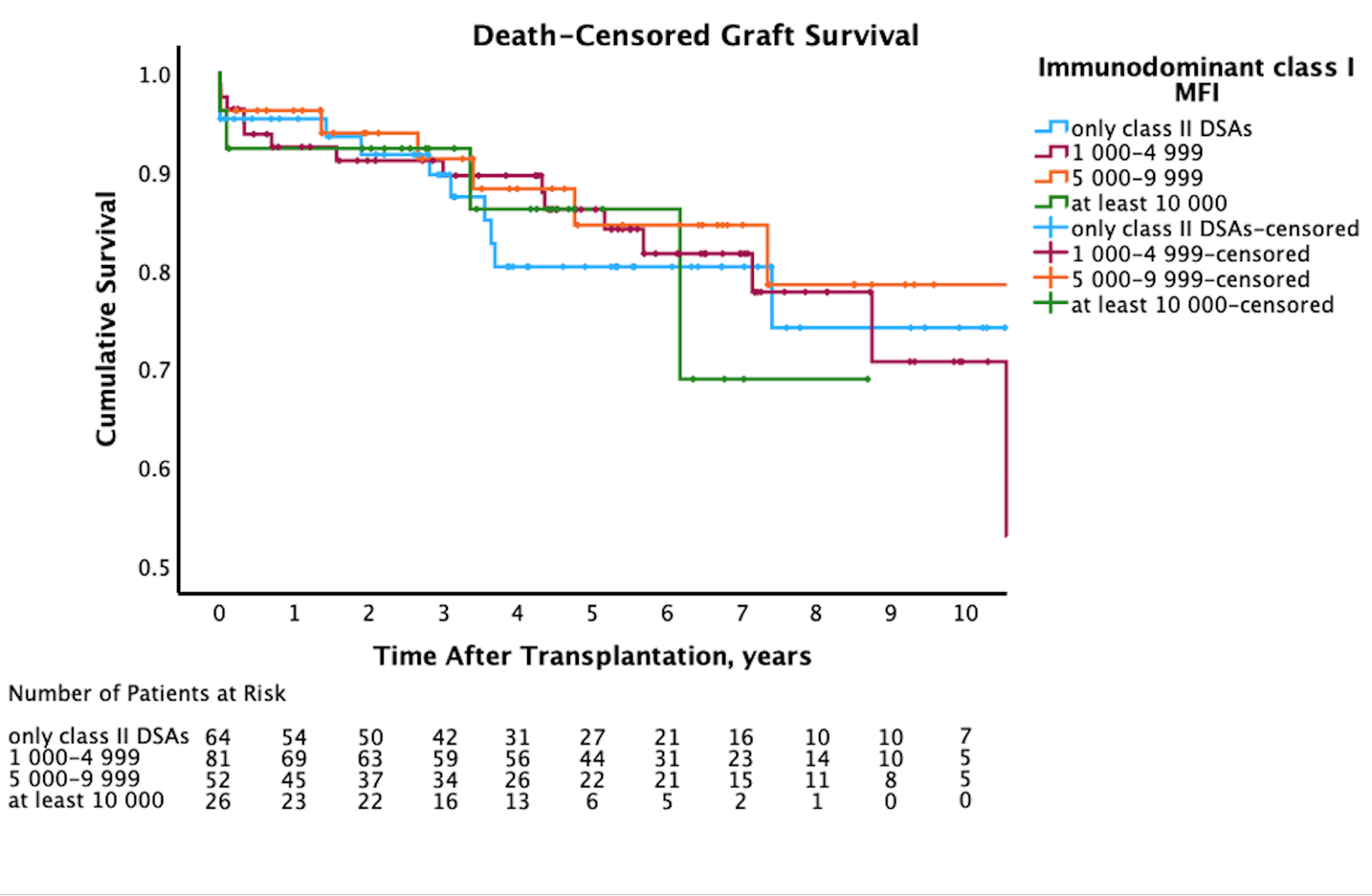

Supplement: Supplementary file 4 [file Image2.tiff]
