# Supplementary material for: Long-term HLA-incompatible kidney transplant outcomes
Source: Transpl Int. 2026 Jul 16;39:16478. doi: 10.3389/ti.2026.16478 (PMC13422208; doi:10.3389/ti.2026.16478)
Supplement: Supplementary file 3 [file Supplementaryfile1.docx]

Table S1. Results of univariate Cox regression analyses for BPARs and AMRs within different comparison groups.

| **Variable** | **BPARs, Hazard ratio (95% CI)** | **BPARs, p-value** | **AMRs, Hazard ratio (95% CI)** | **AMRs, p-value** |
| --- | --- | --- | --- | --- |
| DSA status  all DSAs (vs no DSAs)  class I DSAs (vs no class I DSAs)  class II DSAs (vs no class II DSAs) | 3.223 (2.572-4.039)  3.065 (2.362-3.977)  4.184 (3.180-5.506) | <.001  <.001  <.001 | 35.121 (22.375-55.127)  21.862 (14.340-33.329)  32.252 (21.118-49.255) | <.001  <.001  <.001 |
| Categorized cumulative MFI (vs MFI 1 000 – 4 999)  5 000-9 999  at least 10 000 | 1.254 (.675-2.327)  2.383 (1.476-3.846) | <.001  .474  <.001 | 2.112 (.916-4.871)  4.558 (2.311-8.991) | <.001  .080  <.001 |
| Categorized cumulative MFI, class I DSAs (vs only class II DSAs)  1 000-4 999  5 000-9 999  at least 10 000 | .637 (.364-1.117)  1.048 (.574-1.911)  1.528 (.865-2.699) | .035  .116  .879  .144 | .508 (.243-1.065)  1.327 (.662-2.657)  1.488 (.733-3.023) | .032  .073  .425  .271 |
| Categorized cumulative MFI, class II DSAs (vs only class I DSAs)  1 000-4 999  5 000-9 999  at least 10 000 | 1.502 (.869-2.596)  2.626 (1.403-4.915)  1.920 (1.110-3.323) | .012  .145  .003  .020 | 1.311 (.605-2.841)  4.125 (1.983-8.583)  3.034 (1.576-5.840) | <.001  .492  <.001  <.001 |

Table S2. Categorized immunodominant MFI: Multivariable Cox regression analysis for DCGS.

| **Variable** | **Hazard Ratio (95% CI)** | **p-value** |
| --- | --- | --- |
| Baseline kidney disease (vs diabetic kidney disease)       Glomerulonephritis       Polycystic kidney disease       Other | 1.054 (.800-1.390)  .615 (.430-.878)  .915 (.691-1.211) | .019  .707  .007  .534 |
| Categorized immunodominant MFI (vs no DSAs)       1000-4999       5000-9999       At least 10 000 | 1.582 (.956-2.617)  1.286 (.595-2.777)  2.386 (1.227-4.638) | .043  .074  .522  .010 |
| Cold ischemia time (for each 1 minute increased) | 1.001 (1.000-1.001) | <.001 |
| Donor age (for each 1 year increased) | 1.025 (1.016-1.034) | <.001 |
| Induction immunosuppression, other than iv steroids (vs no) | .783 (.567-1.083) | .140 |
| On cyclosporine (vs tacrolimus) | 1.251 (.963-1.625) | .093 |
| Recipient age (for each 1 year increased) | .985 (.977-.993) | <.001 |
| Recipient sex (vs female) | .940 (.757-1.167) | .576 |
| Retransplantation (vs no) | 1.427 (1.009-2.017) | .044 |
| Time on the waitlist (for each 1 day increase) | 1.000 (1.000-1.000) | .243 |

Table S3. Results of univariate Cox regression analysis for BPARs and AMRs within different immunodominant MFI levels.

| **Variable** | **BPARs, Hazard Ratio (95% CI)** | **BPARs, p-value** | **AMRs, Hazard ratio (95%, CI)** | **AMRs, p-value** |
| --- | --- | --- | --- | --- |
| Categorized immunodominant MFI (vs no DSAs)  1 000-4 999  5 000-9 999  at least 10 000 | 1.687 (1.190-2.393)  2.359 (1.533-3.629)  2.101 (1.463-3.016) | <.001  .003  <.001  <.001 | 13.105 (6.954-24.695)  36.294 (19.769-66.633)  27.641 (15.622-48.906) | <.001  <.001  <.001  <.001 |
| Categorized immunodominant MFI, class I DSAs (vs no class I DSAs)  1 000-4 999  5 000-9 999  at least 10 000 | 2.164 (1.454-3.222)  2.846 (1.828-4.431)  1.776 (1.078-2.927) | <.001  <.001  <.001  .024 | 13.530 (7.291-25.107)  27.086 (15.113-48.542)  11.443 (5.540-23.636) | <.001  <.001  <.001  <.001 |
| Categorized immunodominant MFI, class II DSAs (vs no class II DSAs)  1 000-4 999  5 000-9 999  at least 10 000 | 1.551 (1.045-2.303)  1.817 (.998-3.306)  2.164 (1.363-3.435) | <.001  .029  .051  .001 | 8.489 (4.567-15.781)  19.903 (10.184-38.895)  20.272 (11.199-36.697) | <.001  <.001  <.001  <.001 |
